# Management of patients with recurrent/metastatic endometrial cancer: Consensus recommendations from an expert panel from Brazil

**DOI:** 10.3389/fonc.2023.1133277

**Published:** 2023-03-09

**Authors:** Diocésio Alves Pinto de Andrade, Andréa Paiva Gadelha Guimarães, Andréia Cristina de Melo, Angélica Nogueira-Rodrigues, Larissa Müller Gomes, Mariana Scaranti, Joyce Maria Lisboa Maia, Alessandra Menezes Morelle, Candice Amorim de Araújo Lima Santos, Cristiano de Pádua Souza, Daniela de Freitas, Donato Callegaro Filho, Eduardo Paulino, Elge Werneck Araújo Júnior, Juliana Martins Pimenta, Marcela Bonalumi dos Santos, Michelle Samora de Almeida, Ronaldo Pereira Souza, Samantha Cabral, Fernando Cotait Maluf

**Affiliations:** ^1^ Brazilian Gynecologic Oncology Group, EVA, São Paulo, Brazil; ^2^ InORP Oncoclínicas Group, Ribeirão Preto, Brazil; ^3^ AC Camargo Cancer Center, São Paulo, Brazil; ^4^ Brazilian Nacional Cancer Institute – INCA, Rio de Janeiro, Brazil; ^5^ Universidade Federal de Minas Gerais, Belo Horizonte, Brazil; ^6^ CPO Oncoclínicas Group, São Paulo, Brazil; ^7^ DASA – Hospital 9 de Julho, São Paulo, Brazil; ^8^ Cancer Treatment Center, MedRadius –, Maceió, Brazil; ^9^ Hospital Moinhos de Vento, Porto Alegre, Brazil; ^10^ Instituto de Medicina Integral Prof. Fernando Figueira (IMIP), Recife, Brazil; ^11^ Barretos Cancer Hospital, Barretos, Brazil; ^12^ Hospital Sírio-Libanês, São Paulo, Brazil; ^13^ Hospital Israelita Albert Einstein, São Paulo, Brazil; ^14^ IHOC Oncoclínicas Group, Curitiba, Brazil; ^15^ Hospital Beneficiência Portuguesa de São Paulo, São Paulo, Brazil; ^16^ Hospital do Coração HCOR Oncologia, São Paulo, Brazil; ^17^ Universidade de São Paulo, São Paulo, Brazil

**Keywords:** endometrial cancer, consensus, Brazil, metastatic, recurrence

## Abstract

**Background:**

Endometrial cancer is of increasing concern in several countries, including Brazil, in part because of an ageing population, declines in fertility, and the increasing prevalence of obesity. Although endometrial tumors had lagged behind other cancer types in terms of treatment improvements, molecular characterization of these tumors is paving the way for novel therapies and an expansion of the therapeutic arsenal. We aimed to help medical oncologists who manage patients with recurrent or metastatic endometrial cancer in the Brazilian healthcare setting.

**Methods:**

The panel, composed of 20 medical oncologists, convened in November 2021 to address 50 multiple-choice questions on molecular testing and treatment choices. We classified the level of agreement among panelists as (1) consensus (≥75% choosing the same answer), (2) majority vote (50% to <75%), or (3) less than majority vote (<50%).

**Results:**

Consensus was present for 25 of the 50 questions, whereas majority vote was present for an additional 23 questions. Key recommendations include molecular testing for every patient with recurrent/metastatic endometrial cancer; choice of first-line treatment according to microsatellite instability and HER2, with the addition of programmed death ligand 1 (PD-L1) and hormone receptors (HRs) for second-line therapy; carboplatin and paclitaxel as the preferred option in first-line treatment of HER2-negative disease, with the addition of trastuzumab in HER2-positive disease; pembrolizumab plus lenvatinib as a key option in second line, regardless of HER2, PD-L1 or HRs; and various recommendations regarding treatment choice for patients with distinct comorbidities.

**Conclusion:**

Despite the existing gaps in the current literature, the vast majority of issues addressed by the panel provided a level of agreement sufficient to inform clinical practice in Brazil and in other countries with similar healthcare environments.

## Introduction

Cancer of the uterine corpus is currently the most frequent gynecological malignancy in the US and the sixth most commonly diagnosed neoplasm in women worldwide, with 417,000 new cases estimated for 2020 (1, 2). Even though incidence rates for uterine cancer vary up to 10-fold across countries, and the highest rates are found in North America and Eastern and Northern Europe, incidence rates have been rising worldwide, and countries with historically lower rates have had the largest proportional increase in incidence (3). Although only 7,840 new cases of tumors of the uterine corpus have been estimated for Brazil in 2023 (4), this country had the third largest average annual percent increase (nearly 5%) in incidence in a recent worldwide survey (3, 5, 6). Reasons for the rising incidence of uterine tumors remain incompletely understood, but an ageing population, declines in the fertility rate and the increasing prevalence of obesity are likely to play a major role (1, 7, 8). Endometrial cancer, which accounts for most neoplasms of the uterine corpus – since uterine sarcomas account for only approximately 3-7% of cases (9) – has a median age at diagnosis of 63 years and a strong association with obesity (10, 11). In fact, the association between obesity and endometrial cancer is stronger than for any other common cancer type, and between 36.5% to 54.9% of all uterine corpus tumors in the US are attributable to obesity across different States in that country (7, 10, 12, 13). The use of unopposed estrogen and tamoxifen are also recognized risk factors for endometrial cancer, and changes in the prevalence of these factors likely play a role in current trends for this disease (8, 10, 11, 14).

Even though survival has improved since the mid-1970s for most common cancer types, neoplasms of the uterine corpus represent an exception, largely because of the lack of major treatment advances over the last few decades (2). In recent years, however, molecular characterization of endometrial tumors has become a key component in treatment decisions for patients with recurrent and metastatic disease, and an increased understanding of the molecular basis for different uterine cancers has paved the way for novel therapies for these patients (8, 10, 15–17). As a result, the practicing oncologist has witnessed the recent expansion of the knowledge base and the therapeutic arsenal against recurrent and metastatic endometrial cancer (15, 18). This is particularly evident in second- and subsequent-line treatment, given recent clinical trials of agents with activity against specific molecular subgroups. To informe decisions in the Brazilian healthcare setting, a panel of experts convened in an attempt to establish consensus recommendations in this country for the management of patients with endometrial cancer that is metastatic at diagnosis or presenting as recurrent disease not amenable to local control. The current article presents the results of that panel.

## Panel composition and methodology

The panel was composed by 20 medical oncologists from Brazil, with expertise in gynecological oncology, and working in institutions representing diverse geographic and socioeconomic settings in this country. The panel was coordinated by a committee composed of three of the current authors (DAPA, APGG and FCM), who prepared the 50 multiple-choice questions addressed by the panel and coordinated its conduct by teleconference in November 2021. To provide their recommendations, panel members were expected to take into account the published scientific literature and their own clinical experience. Recommendations were provided in an anonymous manner using an online system that also allowed tabulation of results after the end of the voting period for each question. The questions aimed to elicit recommendations regarding molecular testing and the choice of treatment for patients with recurrent or metastatic endometrial cancer, with particular emphasis on treatment past the first line.

The results for each of the 50 questions addressed by the panel were analyzed descriptively. The level of agreement among voters was ascertained by classifying responses to each question as (1) consensus, (2) majority vote, or (3) less than majority vote. If at least 75% of the voting panel members provided a particular recommendation, consensus was present. If between 50% and 74.9% of the voting members provided a particular recommendation, this was considered as majority vote. For each question, voters had the option to abstain when they felt impeded to provide a qualified response for any reason; of note, recommendation percentages included the option “abstain” in their denominator. The panel was made possible by an educational grant from Merck, Sharp & Dohme, who had no influence on the creation of the questions, the panel conduct, or the writing of the article, all of which rest under the entire responsibility of the authors. Approval by an ethics committee was not required, given the nature of this specific manuscript that only involved expert contributors (the authors). No human subjects were involved.

## Panel recommendations

### Patient assessment before treatment

**Table 1** displays results pertaining to the nine questions related to patient assessment. Consensus was reached for two of those questions: (1) every patient with recurrent/metastatic endometrial cancer should undergo molecular testing before treatment initiation; and (2) computed tomography scan of the chest and abdomen are recommended for baseline assessment before treatment. Moreover, majority vote was present for five of the remaining seven questions: (1) 50% of panelists recommended a more complete histopathological and molecular assessment that includes tumor grade, histological subtype, and the status of microsatellite instability (MSI), HER2, p53, programmed death ligand 1 (PD-L1), polymerase epsilon (POLE), and hormone receptors; (2) 64.7% of panelists recommended that HER2 assessment is necessary in specific cases (and an additional 29.4% recommend it for all cases); (3) 52.9% of panelists recommended against testing for PD-L1; (4) 58.8% did not recommend the assessment of tumor mutational burden (although 41.2% of voters recommended it in specific circumstances); and (5) 52.9% of panelists recommended CA125 assessment. Finally, there was more heterogeneity for the two remaining questions. However, if response options are pooled, 73.4% of voters recommended the assessment of MSI “always”, “mostly” or “in some specific cases”. Likewise, a total of 83.4% of voters recommended the assessment of hormone receptors “always”, “mostly” or “in some specific cases”. Of note, there were no abstentions for questions related to patient assessment.

**Table 1:** Questions related to patient assessment before treatment initiation.

Questions	Recommendations							
Should every patient with metastatic endometrial cancer have molecular analysis before treatment initiation?	Yes	No	Abstain					
	88.2%	11.8%	0%					
Which information is essential in the histopathological report of a patient with metastatic endometrial cancer?	Grade and histological subtype	Grade, histological subtype, and MSI	Grade, histological subtype, MSI, and HER2	Grade, histological subtype, MSI, HER2. and p53	Grade, histological subtype, MSI, HER2, p53, and PD-L1	Grade, histological subtype, MSI, HER2, p53, PD-L1, and POLE	Grade, histological subtype, MSI, HER2, p53, PD-L1, POLE, and HR	Abstain
	0%	0%	33.3%	16.7%	0%	0%	50%	0%
The assessment of MSI is necessary before starting treatment for metastatic endometrial cancer:	Always	Mostly	In some specific cases	Only after failure of first-line chemotherapy	Never	Abstain		
	46.7%	20%	6.7%	26.7%	0%	0%		
The assessment of HER2 is necessary before starting treatment for metastatic endometrial cancer:	Always	Mostly	In some specific cases	Only after failure of first-line chemotherapy	Never	Abstain		
	29.4%	5.9%	64.7%	0%	0%	0%		
The assessment of PD-L1 is necessary before starting treatment for metastatic endometrial cancer:	Always	Mostly	In some specific cases	Only after failure of first-line chemotherapy	Never	Abstain		
	5.9%	11.8%	11.8%	17.6%	52.9%	0%		
The assessment of tumor mutational burden is necessary before starting treatment for metastatic endometrial cancer:	Always	Mostly	In some specific cases	Only after failure of first-line chemotherapy	Never	Abstain		
	0%	0%	41.2%	0%	58.8%	0%		
The assessment of HR is necessary before starting treatment for metastatic endometrial cancer:	Always	Mostly	In some specific cases	Only after failure of first-line chemotherapy	Only after failure of second-line chemotherapy and immunotherapy	Never	Abstain	
	27.8%	27.8%	27.8%	0%	11.1%	5.6%	0%	
The assessment of CA125 is necessary before starting treatment for metastatic endometrial cancer:	Yes	No	Abstain					
	52.9%	47.1%	0%					
What imaging tests are needed to start the treatment of metastatic endometrial cancer?	Chest radiography and abdominal ultrasound	Chest and abdominal CT scan	Chest and abdominal CT scan plus bone scintigraphy	PET-CT scan	Abstain			
	0%	88.9%	11.1%	0%	0%			

CT, computed tomography; HR, hormone receptors; MSI, microsatellite instability; PD-L1, programmed cell death ligand 1; PET, positron-emission tomography.

### First-line treatment

As shown in **Table 2**, consensus was reached for four of six questions pertaining to first-line treatment: (1) first-line treatment should differ according to MSI and HER2 statuses; (2) chemotherapy should be used in first-line treatment of HER2-negative disease; (3) chemotherapy plus trastuzumab should be used in first-line treatment of HER2-positive disease; and (4) the conventional regimen of carboplatin (area under the curve 5-6) and paclitaxel (175 mg/m^2^ every 3 weeks) should be used in the first-line treatment of HER2-negative disease. Majority vote was reached for the remaining two questions: (1) 56.3% of panelists recommended that chemotherapy should not be used alone in first-line treatment of HER2-positive disease; and (2) 66.6% of voters recommended six as the standard number of cycles for first-line treatment.

**Table 2:** Questions related to first-line treatment.

Questions	Recommendations							
Should first-line treatment differ according to MSI and HER2 status?	Yes	No	Abstain					
	88.9%	11.1%	0%					
In which scenario should isolated chemo not be indicated?	MSI-high	PD-L1 positive	HER2 positive	Isolated chemo should always be indicated	Abstain			
	12.5%	0%	56.3%	18.8%	12.5%			
What should be the first-line treatment for HER2-negative metastatic disease?	Chemo	Chemo + pembrolizumab	Chemo + TTZ	CPI	Pembrolizumab + lenvatinib	Hormone therapy	Abstain	
	83.3%	0%	5.6%	0%	5.6%	5.6%	0%	
What should be the first-line treatment for HER2-positive metastatic disease?	Chemo	Chemo + pembrolizumab	Chemo + TTZ	TTZ monotherapy	CPI	Pembrolizumab + lenvatinib	Hormone therapy	Abstain
	12.5%	0%	87.5%	0%	0%	0%	0%	0%
What chemo regimen should be indicated in the first-line treatment of HER2-negative disease?	Carboplatin, AUC 5-6 + paclitaxel, 175 mg/m^2^ every 3 weeks	Carboplatin, AUC 5-6 on Day 1 + paclitaxel, 80 mg/m^2^ D1-D8-D15 every 3 weeks	Carboplatin, AUC 5-6 on Day 1 + paclitaxel, 60 mg/m^2^ D1-D8-D15 every 3 weeks	Platin doublet with another agent (liposomal doxorubicin, gemcitabine)	Platin doublet + bevacizumab	Abstain		
	100%	0%	0%	0%	0%	0%		
How many cycles of chemo should be given in first-line treatment?	4 cycles	6 cycles	8 cycles	Until disease progression and/or limiting toxicity	Abstain			
	0%	66.7%	0%	33.3%	0%			

AUC, area under the curve; Chemo, chemotherapy; CPI, checkpoint inhibitor; MSI, microsatellite instability; PD-L1, programmed cell death ligand 1; TTZ, trastuzumab.

### Second-line treatment

**Figure 1:**
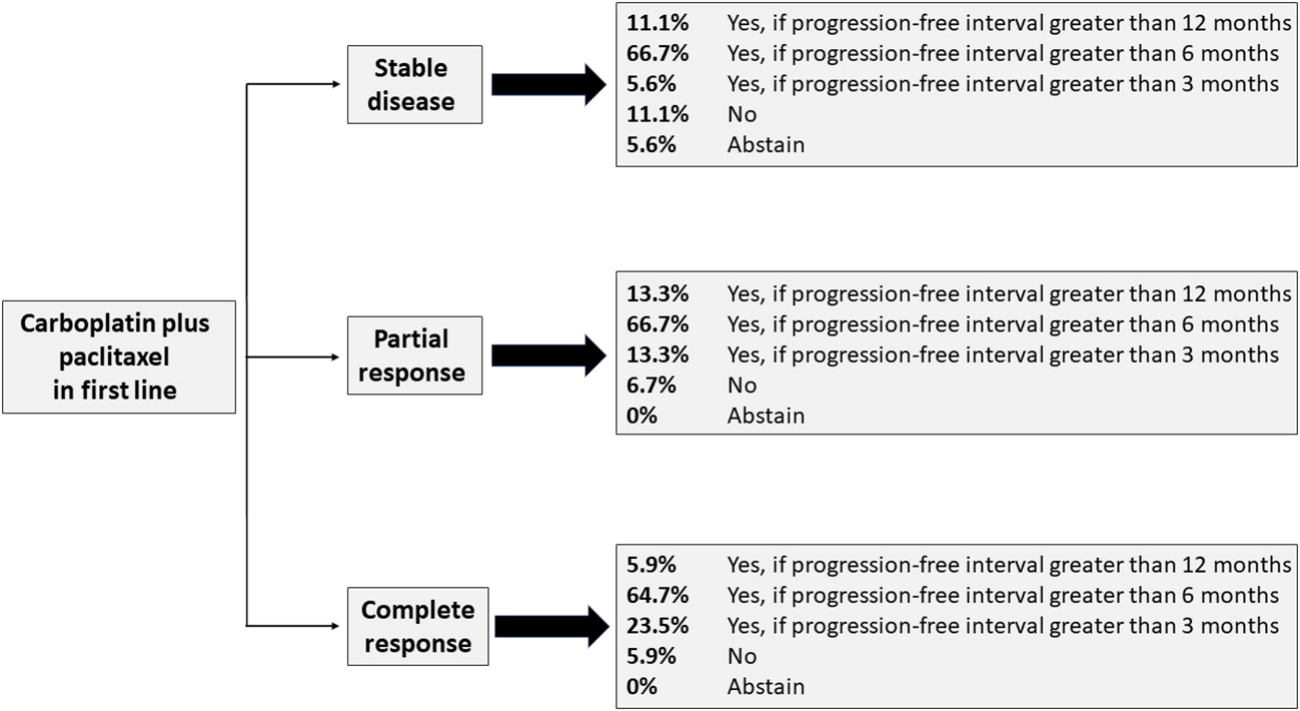
Recommendations for re-exposure to carboplatin and paclitaxel according to response to first-line therapy.

A total of 33 questions addressed issues related to second-line treatment. The first three of these questions have their corresponding recommendations shown pictorially in **Figure 1**, whereas the remaining 30 questions are shown in **Table 3**. Although the three questions depicted in **Figure 1** had only majority vote for one single answer, in all three cases two answers can be pooled to establish a consensus minimum duration of progression-free interval of 6 months before patients are re-exposed to carboplatin and paclitaxel after having stable disease or partial response to first-line treatment, and of 3 months after complete response.

**Table 3:** Questions related to second-line treatment.

Questions	Recommendations									
The assessment of HR is necessary before second-line treatment:	Always	Mostly	In some specific cases	Only after second-line chemo + immunotherapy	Never	Abstain				
	11.1%	33.3%	44.4%	5.6%	5.6%	0%				
The assessment of PD-L1 is necessary before second-line treatment:	Always	Mostly	In some specific cases	Only after second-line chemo + immunotherapy	Never	Abstain				
	11.1%	5.6%	22.2%	0%	55.6%	5.6%				
The assessment of MSI is necessary before second-line treatment:	Always	Mostly	In some specific cases	Only after second-line chemo + immunotherapy	Never	Abstain				
	77.8%	11.1%	5.6%	0%	5.6%	0%				
The assessment of HER2 is necessary before second-line treatment:	Always	Mostly	In some specific cases	Only after second-line chemo + immunotherapy	Never	Abstain				
	16.7%	11.1%	55.6%	0%	16.7%	0%				
The assessment of TMB is necessary before second-line treatment:	Always	Mostly	In some specific cases	Only after second-line chemo + immunotherapy	Never	Abstain				
	5.6%	11.1%	55.6%	0%	27.8%	0%				
Should second-line treatment differ according to MSI, HER2, PD-L1 and HR status?	Yes	No	Abstain							
	94.4%	5.6%	0%							
What should be the second-line treatment for HER2-positive disease without MSI after carboplatin + paclitaxel?	Chemo	Chemo + pembro	Chemo + lenva	Chemo + TTZ	CPI	Pembro + lenva	Lenva monothterapy	TTZ monotherapy	Hormone therapy	Abstain
	5.6%	0%	0%	27.8%	0%	61.1%	0%	0%	0%	5.6%
What should be the second-line treatment for HER2-positive disease without MSI after carboplatin + paclitaxel + trast?	Chemo	Chemo + Pembro	TDM-1	Chemo + lenva	CPI	Pembro + lenvatinib	Lenva monothterapy	Hormone therapy	Abstain	–
	0%	0%	5.6%	0%	0%	94.4%	0%	0%	0%	
What should be the second-line treatment for HER2-negative disease without MSI after carboplatin + paclitaxel?	Pembro 200 mg every 3 weeks	Pembro 200 mg every 3 weeks + lenva 20 mg/day	Pembro 200 mg every 3 weeks + lenva 14 mg/day	Pembro 200 mg every 3 weeks + lenva 10 mg/day	Lenva 20 mg/day monotherapy	Abstain				
	0%	86.7%	0%	13.3%	0%	0%				
Is the choice of second-line treatment with pembro + lenva influenced by HR status?	Yes	No	Abstain							
	5.6%	94.4%	0%							
									
Is the choice of second-line treatment with pembro + lenva influenced by HER2 status?	Yes	No	Abstain							
	17.6%	82.4%	0%							
Is the choice of second-line treatment with pembro + lenva influenced by MSI status?	Yes	No	Abstain							
	78.9%	21.1%	0%							
Is the choice of second-line treatment with pembro + lenva influenced by PD-L1 status?	Yes	No	Abstain							
	5.6%	94.4%	0%							
Is the choice of second-line treatment with pembro + lenva influenced by histological subtype?	Yes, only for endometrioid carcinomas	Yes, only for serous carcinomas	Yes, only for clear-cell carcinomas	Yes, only for carcinosarcomas	No	Abstain				
	11.1%	0%	0%	5.6%	72.2%	11.1%				
Is heart disease a contraindication to second-line treatment with pembro + lenva?	Yes	No	Abstain							
	23.5%	58.8%	17.6%							
Is hypertension a contraindication to second-line treatment with pembro + lenva?	Yes	No	Abstain							
	11.1%	88.9%	0%							
Is diabetes mellitus a contraindication to second-line treatment with pembro + lenva?	Yes	No	Abstain							
	0%	100%	0%							
Is dyslipidemia a contraindication to second-line treatment with pembro + lenva?	Yes	No	Abstain							
	0%	100%	0%							
Is there contraindication to second-line treatment with pembro + lenva according to performance status?	Yes	No	Abstain							
	77.8%	22.2%	0%							
What should be the second-line treatment for HER2- positive disease with MSI after carboplatin + paclitaxel?	Chemo	Chemo + pembrolizumab	Chemo + lenva	Chemo + trast	CPI	Pembro + lenva	Lenva monothterapy	Hormone therapy	Abstain	
	0%	0%	0%	15.8%	68.4%	15.8%	0%	0%	0%	
What should be the second-line treatment for HER2- positive disease with MSI after carboplatin + paclitaxel + trast?	Chemo	Chemo + pembrolizumab	TDM-1	Chemo + lenva	CPI	Pembro + lenvatinib	Lenva monothterapy	Hormone therapy	Abstain	
	0%	0%	0%	0%	72.2%	27.8%	0%	0%	0%	
What should be the second-line treatment for HER2- negative disease with MSI after carboplatin + paclitaxel?	Chemo	Chemo + pembrolizumab	Chemo + lenva	CPI	Pembro + lenva	Lenva monothterapy	Hormone therapy	Abstain		
	0%	0%	0%	78.9%	21.1%	0%	0%	0%		
Is the choice of second-line treatment with CPI influenced by HR status?	Yes	No	Abstain							
	5.6%	94.4%	0%							
Is the choice of second-line treatment with CPI influenced by HER2 status?	Yes	No	Abstain							
	11.1%	88.9%	0%							
Is the choice of second-line treatment with CPI influenced by MSI status?	Yes	No	Abstain							
	94.7%	5.3%	0%							
Is the choice of second-line treatment with CPI influenced by PD-L1 status?	Yes	No	Abstain							
	15.8%	84.2%	0%							
Is the choice of second-line treatment with CPI influenced by TMB?	Yes	No	Abstain							
	47.4%	52.6%	0%							
Is the choice of second-line treatment with CPI influenced by histological subtype?	Yes, only for endometrioid carcinomas	Yes, only for serous carcinomas	Yes, only for clear-cell carcinomas	Yes, only for carcinosarcomas	No	Abstain				
	0%	0%	0%	5.6%	88.9%	5.6%				
Can pembro in second-line treatment with or without lenva be modified from 200 mg every 3 weeks to 400 mg every 6 weeks?	Yes	No	Abstain							
	38.9%	50%	11.1%							
Should lenva combined with pembro as part of second-line treatment be routinely started at doses <20 mg/day?	Yes	No	Abstain							
	47.1%	52.9%	0%							

Chemo, chemotherapy; CPI, checkpoint inhibitor; HR, hormone receptor; Lenva, lenvatinib; MSI, microsatellite instability; PD-L1, programmed cell death ligand 1; Pembro, pembrolizumab; TDM-1, trastuzumab emtansine; TMB, tumor mutational burden; TTZ, trastuzumab.

As shown in **Table 3**, consensus was reached for 18 of the remaining 30 questions pertaining to second-line treatment: (1) the assessment of MSI is necessary before second-line treatment; (2) the choice of second-line treatment should be guided by MSI, HER2, PD-L1 and hormone receptor status; (3) pembrolizumab plus lenvatinib should be the treatment of choice for HER2-positive disease without MSI after the regimen of carboplatin, paclitaxel and trastuzumab; (4) pembrolizumab, 200 mg every 3 weeks, plus lenvatinib, 20 mg/day, should be the second-line treatment for HER2-negative disease without MSI after carboplatin plus paclitaxel; the choice of second-line treatment with pembrolizumab plus lenvatinib should not be influenced by (5) hormone receptor status, (6) HER2 status or (7) PD-L1 status, but (8) should be influenced by MSI status; neither (9) hypertension nor (10) diabetes mellitus or (11) dyslipidemia are contraindications for second-line treatment with pembrolizumab plus lenvatinib, but (12) poor performance status is; (13) second-line treatment for HER2-negative disease with MSI after carboplatin plus paclitaxel should be an immune checkpoint inhibitor; the choice of second-line treatment with a checkpoint inhibitor should not be influenced by (14) hormone receptor status, (15) HER2 status, (16) PD-L1 status or (17) histological subtype, but (18) should be guided by MSI status.

Majority vote was present for 11 of 30 questions related to second-line treatment, whereas for one question there was less than majority vote (see **Table 3**). It should be noted, however, that for some of these questions the response options can be pooled in a manner that indicates a clear preference for somewhat similar interventions. This is the case, for example, of the need to assess hormone receptors before second-line treatment: 11.1% of panelists indicated that this should always be done, 33.3% that this should be done in most cases, and 44.4% that this should be done in specific cases. On the other hand, some questions with a majority vote indicated a clear dichotomy of opinions, such as whether the choice of second-line treatment with an immune checkpoint inhibitor should be influenced by tumor mutational burden; in this case, 47.4% of voters believe the choice would depend on that assessment, whereas 52.6% believe that it would not.

### Treatment past two lines

Two questions addressed treatment beyond two lines. For the first, about what should be the choice after first-line treatment with carboplatin plus paclitaxel and second-line treatment with pembrolizumab plus lenvatinib, 82.4% of panelists recommended chemotherapy regimens other than carboplatin plus paclitaxel. For the question of how many lines of chemotherapy should a patient receive, regardless of histological subtype or biomarkers, 73.7% of voters indicated that the number of lines should not be fixed but rather guided by the continued presence of adequate performance status.

## Discussion

To our knowledge, this is the first consensus meeting conducted in Brazil to address the management of advanced endometrial cancer. Previous work from our country has successfully addressed the surgical management of this disease, which is of increasing concern in many countries (3, 19). As many other middle-income countries, the healthcare system in Brazil is subject to accessibility constraints, and local practice guidelines are relatively scarce. We believe the current work can help medical oncologists to make decisions and base their practice on consensus recommendations. Nevertheless, consensus was present for only 25 of the 50 questions, in many cases as a reflection of current doubts in the literature. On the other hand, majority vote was present for an additional 23 questions, and less than majority vote was present for only three questions. Therefore, for the vast majority of issues addressed by the panel, there seems to be a sufficient level of agreement among members to guide clinical practice.

The classification of endometrial tumors has been predominantly based on morphology, with increasing use of ancillary testing, such as immunohistochemistry. Moreover, molecular subtyping increasingly provides prognostic insight and helps the practicing clinician in making treatment decisions (8, 10, 17, 20). Importantly, randomized trials – such as Postoperative Radiation Therapy in Endometrial Cancer (PORTEC) 4a and the RAINBO trials program – are ongoing to assess the validity of making adjuvant treatment decisions for early-stage disease based on molecular classification (21, 22). In the advanced-disease setting, molecular tumor features can already be used to guide therapy, particularly in uterine serous cancers and MSI tumors (10, 23–26). The vast majority of panel members consider the assessment of MSI and HER2 as essential for all patients before treatment initiation, with division of opinions about other markers, such as tumor mutational burden, p53, PD-L1, POLE, and hormone receptors (**Table 1**). Likewise, there is a predominant view that molecular markers, particularly MSI and HER2, should guide second-line therapy (**Table 2**). It should be noted that POLE mutations are identified with the use of sequencing, which may not be widely available. POLE and p53 are important to define molecular classification, but they do not yet influence the choice of treatment (27). PD-L1 proved to be a predictor of response in other tumors, such as lung cancer (28), but it did not influence therapeutic responses with immunotherapy in the treatment of endometrial cancer (29). Furthermore, the assessment of tumor mutational burden may be restricted to large referral centers, and up to now it has not been shown to influence treatment choices or prognostic assessment among patients with endometrial cancer (30).

The current results indicate consensus among panel members that patients with HER2-positive tumors benefit from the addition of trastuzumab to first-line therapy with carboplatin and paclitaxel. Even though the evidence in the literature is restricted to uterine serous tumors (15, 23), and based on a phase 2 trial with 61 patients, the current panel indicates HER2 testing as an essential step in patients who are candidates to first-line therapy and a preference for adding trastuzumab to such treatment, thus increasing progression-free survival (**Table 2**) (23). For all other endometrial tumors, the literature suggests, and the panel indicates by consensus, that carboplatin plus paclitaxel remains the standard of care in the first line, with a median progression-free survival of 13 months and overall survival of 37 months (31). Of note, recent results indicate that the regimen of carboplatin plus paclitaxel is also the standard of care for uterine carcinosarcoma (32).

Re-exposure to carboplatin plus paclitaxel is indicated by the panel – and corroborated by a retrospective study (33) – when the progression-free interval is at least 6 months, but a shorter interval is considered appropriate for patients with a complete response to first-line therapy (**Figure 1**). Indeed, the panel recommends by consensus that chemotherapy regimens other than carboplatin plus paclitaxel be used after failure of first-line treatment with this regimen and failure of second-line treatment with pembrolizumab plus lenvatinib. As suggested by several of the panel recommendations for second-line therapy, the latter regimen seems to be the currently preferred option in second line for patients without MSI, even for those with HER2-positive disease, regardless of previous treatment with trastuzumab (**Table 2**). In KEYNOTE-775, a phase 3 trial, the combination of pembrolizumab plus lenvatinib improved the objective response rate, progression-free survival, and overall survival, regardless of MSI status, when compared with chemotherapy of physician’s choice in patients with one or two prior lines of therapy (34), thus confirming results from a previous single-arm trial (KEYNOTE-146) (29, 35). The latter results led to the approval of this combination in several countries, including Brazil, and to the design of an ongoing phase 3 trial in the first line, in comparison with carboplatin plus paclitaxel (36).

The panel addressed issues related to the toxicity of the combination of pembrolizumab plus lenvatinib and expressed greater concern with its use in patients with poor performance status or with heart disease; on the other hand, there is little concern for patients with hypertension, dyslipidemia, or diabetes mellitus (**Table 3**). The toxicity associated with this combination includes hypertension, fatigue, nausea/vomiting, diarrhea, decreased appetite, weight loss, hypothyroidism, hand-foot syndrome, musculoskeletal pain, stomatitis, and proteinuria (34, 35, 37). These adverse reactions may usually be managed with supportive care medications and judicious lenvatinib dose modifications (37).

As in other tumor types, the role of immunotherapy is expanding in endometrial cancer. Single-agent checkpoint inhibitors are also an option among patients with disease progression after the first line. Pembrolizumab was approved in 2017 for patients with mismatch repair deficiency or MSI-high tumors (including endometrial cancer), based on aggregate results from five single-arm trials (38). Subsequent results from the single-arm trial, KEYNOTE-158, among patients with previously treated, advanced endometrial cancer with mismatch repair deficiency or MSI-high, have shown an objective response rate of 48%, median progression-free survival of 13.1 months, and median overall survival that was not reached at the time of reporting (24). Other checkpoint inhibitors are under investigation for advanced endometrial cancer, and these include dostarlimab, recently approved in the US for recurrent, mismatch repair deficiency tumors based on results from the ongoing GARNET trial (26). Of note, the panel indicated a preference, at least by majority vote, for the use of a checkpoint inhibitor as the preferred option for second-line treatment of HER2-negative or HER2-positive disease with MSI, whether or not trastuzumab has been used in the first line (**Table 3**).

There seems to be no strong preference for the use of hormone therapy by the current panel, considering the settings investigated and the questions posed to members. Nevertheless, it should be noted that patients with advanced or recurrent endometrioid endometrial tumors with low-volume disease and a long disease-free interval, especially if they have insufficient conditions for chemotherapy, can be treated with progesterone; this is even more important for tumors that are positive for hormone receptors, particularly if they are grade 1 or 2, even though no randomized trials have compared this approach versus chemotherapy in the first line (10, 15). Likewise, hormone therapy can be an option for patients with more limited performance status or for treatment past the first line in selected cases.

Despite its findings and potential relevance for practicing clinicians, our consensus has some limitations, including (1) the fact that not all 20 physicians answered the 50 questions asked, (2) the need to rely on scientific literature with lower level of evidence and/or grades of recommendation (e.g., phase 2 trials) to define consensus regarding some of the settings for which no higher level exists, and (3) the amalgamation of recommendations without a distinction between public and private healthcare settings in our country.

## Conclusion

Given that at least majority vote was present for 47 of the 50 questions addressed by the panel, we believe that the current work can help medical oncologists treating patients with endometrial cancer in Brazil and in other countries with similar healthcare environments to make decisions informed by the current recommendations, which are based on the scientific literature and expert opinion. Nevertheless, several questions regarding the management of these patients remain, either because of knowledge gaps in the literature or because some topics have not been addressed by the current panel. Therefore, continued effort is needed to ensure adequate dissemination and implementation of current best practices in this and other fields in oncology.

## Data availability statement

The original contributions presented in the study are included in the article/**Supplementary Material**. Further inquiries can be directed to the corresponding author.

## Author contributions

All authors contributed equally to the development of the project and the writing of the paper. The panel was coordinated by a committee composed of three of the current authors (DA, AG and FM). All authors contributed to the article and approved the submitted version.
