# Supplementary material for: Management of patients with recurrent/metastatic endometrial cancer: Consensus recommendations from an expert panel from Brazil
Source: Front Oncol. 2023 Mar 9;13:1133277. doi: 10.3389/fonc.2023.1133277 (PMC10033867; doi:10.3389/fonc.2023.1133277)
Supplement: Supplementary file 1 [file DataSheet_1.pdf]

Results of Brazilian Consensus Recommendations about management of patients with Recurrent/Metastatic Endometrial Cancer

Every patient with metastatic endometrial cancer **must** have molecular analysis done before starting treatment?

1	Yes	88.2%
2	No	11.8%
3	Abstain	0%

Which is essential in histopathological report of the patient with metastatic endometrial cancer?

1	Grade and histological subtype	0%
2	Grade, histological subtype and microsatellite instability	0%
3	Grade, histological subtype, microsatellite instability and HER2	33.3%
4	Grade, histological subtype, microsatellite instability, HER2 and p53	16.7%
5	Grade, histological subtype, microsatellite instability, HER2, p53 and PD-L1	0%
6	Grade, histological subtype, microsatellite instability, HER2, p53, PD-L1 and POLE	0%
7	Grade, histological subtype, microsatellite instability, HER2, p53, PD-L1, POLE and hormone receptors	50.0%
8	Abstain	0%

Assessment of **microsatellite instability** is necessary before start the treatment of metastatic endometrial cancer?

1	Always	46.7%
2	Mostly	20.0%
3	In some specific cases	6.7%
4	Only after failure in 1st-line chemotherapy treatment	26.7%
5	Never	0%
6	Abstain	0%

Assessment of **HER2** is necessary before start the treatment of metastatic endometrial cancer?

1	Always	29.4%
2	Mostly	5.9%
3	In some specific cases	64.7%
4	Only after failure in 1st-line chemotherapy treatment	0%
5	Never	0%
6	Abstain	0%

Assessment of **PD-L1** is necessary before start the treatment of metastatic endometrial cancer?

1	Always	5.9%
2	Mostly	11.8%
3	In some specific cases	11.8%
4	Only after failure in 1st-line chemotherapy treatment	17.6%
5	Never	52.9%
6	Abstain	0%

Assessment of **TMB (tumor mutation burden)** is necessary before start the treatment of metastatic endometrial cancer?

1	Always	0%
2	Mostly	0%
3	In some specific cases	41.2%
4	Only after failure in 1st-line chemotherapy treatment	0%
5	Never	58.8%
6	Abstain	0%

Assessment of **hormone receptors** is necessary before start the treatment of metastatic endometrial cancer?

1	Always	27.8%
2	Mostly	27.8%
3	In some specific cases	27.8%
4	Only after failure in 1st-line chemotherapy treatment	0%
5	Only after failure in 2nd-line chemotherapy and imunotherapy treatment	11.1%
6	Never	5.6%
7	Abstain	0%

Assessment of **CA 125 antigen** is necessary before start the treatment of metastatic endometrial cancer?

1	Yes	52.9%
2	No	47.1%
3	Abstain	0%

What **imagin tests** are needed to start the treatment of metastatic endometrial cancer?

1	Chest radiography and abdominal ultrasound	0%
2	Chest and abdominal CT scan	88.9%
3	Chest and abdominal CT scan; bone scintigraphy	11.1%
4	PET-CT scan	0%
5	Abstain	0%

Is there any difference in 1st-line treatment according to specific groups of metastatic endometrial cancer (**microssatelite instability; HER2**)?

1	Yes	88.9%
2	No	11.1%
3	Abstain	0%

Is which scenario isolated chemotherapy treatment would not be indicated in 1st-line for metastatic endometrial cancer?

1	Microssatelite instability-high	12.5%
2	PD-L1 positive	0%
3	HER2 positive	56.3%
4	Never, but isolated chemotherapy is the 1st-line treatment ever	18.8%
5	Abstain	12.5%

Is which scenario **chemotherapy** treatment **would not be indicated** in 1st-line for metastatic endometrial cancer?

1	Microssatelite instability-high	18.8%
2	PD-L1 positive	0%
3	HER2 positive	6.3%
4	Never, but isolated chemotherapy is the 1st-line treatment ever	56.3%
5	Abstain	18.8%

What is the 1st-line treatment for **HER2 negative** metastatic endometrial cancer?

1	Chemotherapy	83.3%
2	Chemotherapy + Pembrolizumab	0%
3	Chemotherapy + Trastuzumab	5.6%
4	Pembrolizumab monotherapy	0%
5	Pembrolizumab + Lenvatinib	5.6%
6	Hormonotherapy	5.6%
7	Abstain	0%

What is the 1st-line treatment for **HER2 positive** metastatic endometrial cancer?

1	Chemotherapy	12.5%
2	Chemotherapy + Pembrolizumab	0%
3	Chemotherapy + Trastuzumab	87.5%
4	Trastuzumab monotherapy	0%
5	Pembrolizumab monotherapy	0%
6	Pembrolizumab + Lenvatinib	0%
7	Hormonotherapy	0%
8	Abstain	0%

What is the chemotherapy regimen indicated in **1st-line** for **HER2 negative** metastatic endometrial cancer?

1	Carboplatin AUC 5-6 + paclitaxel 175 mg/m2 every 3 weeks	100.0%
2	Carboplatin AUC 5-6 D1 + paclitaxel 80 mg/m2 D-D8-D15 every 3 weeks	0%
3	Carboplatin AUC 5-6 D1 + paclitaxel 60 mg/m2 D-D8-D15 every 3 weeks	0%
4	Platin doublet with another chemotherapy (liposomal doxorubicin; gemcitabine)	0%
5	Platin doublet + Bevacizumab	0%
6	Abstain	0%

How many cycles of chemotherapy is indicated in **1st-line** metastatic endometrial cancer setting?

1	4 cycles	0%
2	6 cycles	66.7%
3	8 cycles	0%
4	Even disease progression and/or limiting toxicity	33.3%
5	Abstain	0%

Patient had **stable disease after 1st-line** chemotherapy treatment. **Re-exposure to carboplatin + paclitaxel** every 3 weeks is na option in case of new relapse?

1	Yes, if progression-free interval greater than 12 months	11.1%
2	Yes, if progression-free interval greater than 6 months	66.7%
3	Yes, if progression-free interval greater than 3 months	5.6%
4	No	11.1%
5	Abstain	5.6%

Patient had **partial response after 1st-line** chemotherapy treatment. **Re-exposure to carboplatin + paclitaxel** every 3 weeks is na option in case of new relapse?

1	Yes, if progression-free interval greater than 12 months	13.3%
2	Yes, if progression-free interval greater than 6 months	66.7%
3	Yes, if progression-free interval greater than 3 months	13.3%
4	No	6.7%
5	Abstain	0%

Patient had **complete response after 1st-line** chemotherapy treatment. **Re-exposure to carboplatin + paclitaxel** every 3 weeks is na option in case of new relapse?

1	Yes, if progression-free interval greater than 12 months	5.9%
2	Yes, if progression-free interval greater than 6 months	64.7%
3	Yes, if progression-free interval greater than 3 months	23.5%
4	No	5.9%
5	Abstain	0%

Are **hormone receptors analysis** necessary in the evaluation of **2nd-line** treatment for metastatic endometrial cancer?

1	Always	11.1%
2	Mostly	33.3%
3	In some specific cases	44.4%
4	Only after 2nd-line chemotherapy + immunotherapy treatment	5.6%
5	Never	5.6%
6	Abstain	0%

Is **PD-L1 analysis** necessary in the evaluation of **2nd-line** treatment for metastatic endometrial cancer?

1	Always	11.1%
2	Mostly	5.6%
3	In some specific cases	22.2%
4	Only after 2nd-line chemotherapy + immunotherapy treatment	0%
5	Never	55.6%
6	Abstain	5.6%

Is **microsatellite instability analysis** necessary in the evaluation of **2nd-line** treatment for metastatic endometrial cancer?

1	Always	77.8%
2	Mostly	11.1%
3	In some specific cases	5.6%
4	Only after 2nd-line chemotherapy + immunotherapy treatment	0%
5	Never	5.6%
6	Abstain	0%

Is **HER2 analysis** necessary in the evaluation of **2nd-line** treatment for metastatic endometrial cancer?

1	Always	16.7%
2	Mostly	11.1%
3	In some specific cases	55.6%
4	Only after 2nd-line chemotherapy + immunotherapy treatment	0%
5	Never	16.7%
6	Abstain	0%

Is **TMB analysis** necessary in the evaluation of **2nd-line** treatment for metastatic endometrial cancer?

1	Always	5.6%
2	Mostly	11.1%
3	In some specific cases	55.6%
4	Only after 2nd-line chemotherapy + immunotherapy treatment	0%
5	Never	27.8%
6	Abstain	0%

Is there any difference in **2nd-line** treatment according to specific groups of metastatic endometrial cancer (**microssatelite instability; HER2; PD-L1; hormone receptors**)?

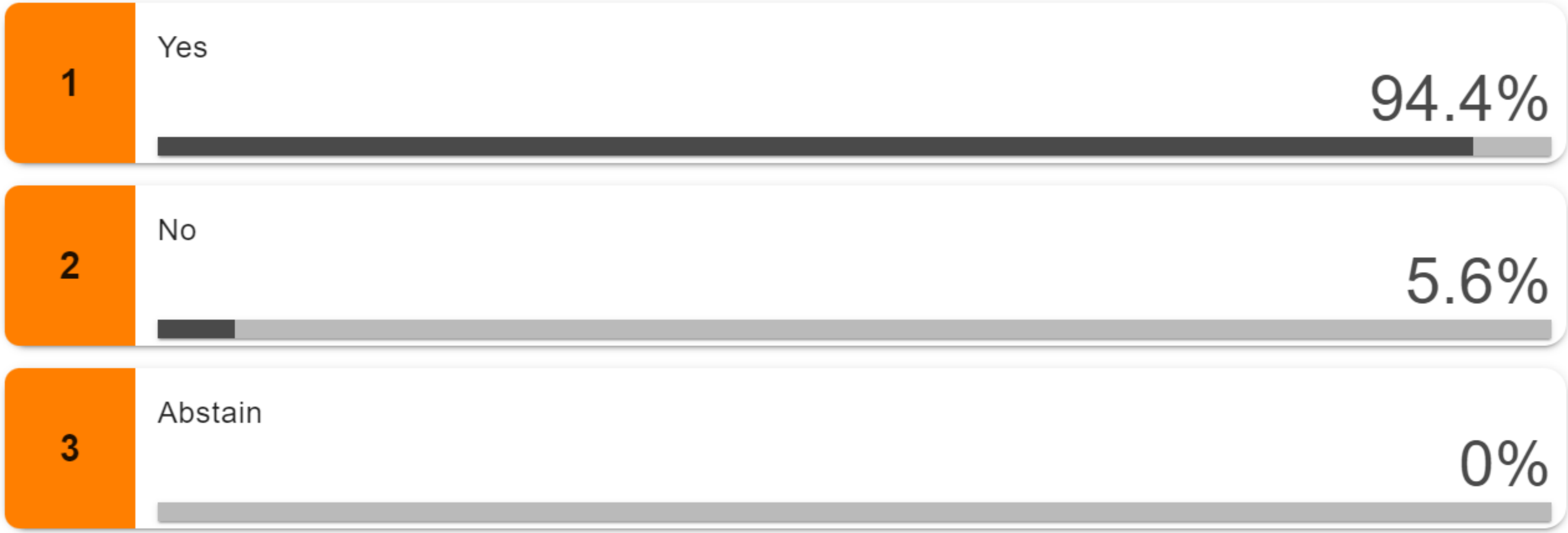

What is the **2nd-line treatment** for metastatic endometrial cancer **HER2 positive without microsatellite instability** after carboplatin + paclitaxel?

1	Chemotherapy	5.6%
2	Chemotherapy + Pembrolizumab	0%
3	Chemotherapy + Lenvantinib	0%
4	Chemotherapy + Trastuzumab	27.8%
5	Pembrolizumab monotherapy	0%
6	Pembrolizumab + Lenvatinib	61.1%
7	Lenvantinib monothterapy	0%
8	Trastuzumab monotherapy	0%
9	Hormonotherapy	0%
10	Abstain	5.6%

What is the **2nd-line treatment** for metastatic endometrial cancer **HER2 positive without microsatellite instability** after carboplatin + paclitaxel + trastuzumab?

1	Chemotherapy	0%
2	Chemotherapy + Pembrolizumab	0%
3	TDM-1	5.6%
4	Chemotherapy + Lenvantinib	0%
5	Pembrolizumab monotherapy	0%
6	Pembrolizumab + Lenvatinib	94.4%
7	Lenvantinib monothterapy	0%
8	Hormonotherapy	0%
9	Abstain	0%

What is the **2nd-line treatment regimen** for metastatic endometrial cancer **HER2 negative without microsatellite instability** after carboplatin + paclitaxel?

1	Pembrolizumab 200 mg every 3 weeks	0%
2	Pembrolizumab 200 mg every 3 weeks + Lenvatinib 20 mg/day	86.7%
3	Pembrolizumab 200 mg every 3 weeks + Lenvatinib 14 mg/day	0%
4	Pembrolizumab 200 mg every 3 weeks + Lenvatinib 10 mg/day	13.3%
5	Lenvatinib 20 mg/day monotherapy	0%
6	Abstain	0%

Is the choice of **2nd-line treatment with Pembrolizumab + Lenvatinib** influenced by the **hormone receptors expression**?

1	Yes	5.6%
2	No	94.4%
3	Abstain	0%

Is the choice of **2nd-line treatment with Pembrolizumab + Lenvatinib** influenced by the **HER2 expression**?

1	Yes	17.6%
2	No	82.4%
3	Abstain	0%

Is the choice of **2nd-line treatment with Pembrolizumab + Lenvatinib** influenced by the **microsatellite instability**?

1	Yes	78.9%
2	No	21.1%
3	Abstain	0%

Is the choice of **2nd-line treatment with Pembrolizumab + Lenvatinib** influenced by the **PD-L1 expression**?

1	Yes	5.6%
2	No	94.4%
3	Abstain	0%

Is the choice of **2nd-line treatment with Pembrolizumab + Lenvatinib** influenced by the **histological subtype**?

1	Yes, only for endometrioid carcinomas	11.1%
2	Yes, only for serous carcinomas	0%
3	Yes, only for clear cell carcinomas	0%
4	Yes, only for carcinosarcomas	5.6%
5	No	72.2%
6	Abstain	11.1%

Is there a contraindication to **2nd-line treatment with Pembrolizumab + Lenvatinib** in patients with **heart disease**?

1	Yes	23.5%
2	No	58.8%
3	Abstain	17.6%

Is there a contraindication to **2nd-line treatment with Pembrolizumab + Lenvatinib** in patients with **hypertension**?

	Option	Result
1	Yes	11.1%
2	No	88.9%
3	Abstain	0%

Is there a contraindication to **2nd-line treatment with Pembrolizumab + Lenvatinib** in patients with **diabetes mellitus**?

1	Yes	0%
2	No	100.0%
3	Abstain	0%

Is there a contraindication to **2nd-line treatment with Pembrolizumab + Lenvatinib** in patients with **dyslipidemia**?

	Option	Result
1	Yes	0%
2	No	100.0%
3	Abstain	0%

Is there a contraindication to **2nd-line treatment with Pembrolizumab + Lenvatinib** according to **ECOG/*performance status***?

1	Yes	77.8%
2	No	22.2%
3	Abstain	0%

What is the **2nd-line treatment** for metastatic endometrial cancer **HER2 positive with microsatellite instability** after carboplatin + paclitaxel?

1	Chemotherapy	0%
2	Chemotherapy + Pembrolizumab	0%
3	Chemotherapy + Lenvantinib	0%
4	Chemotherapy + Trastuzumab	15.8%
5	Pembrolizumab monotherapy	68.4%
6	Pembrolizumab + Lenvatinib	15.8%
7	Lenvantinib monothterapy	0%
8	Hormonotherapy	0%
9	Abstain	0%

What is the **2nd-line treatment** for metastatic endometrial cancer **HER2 positive with microsatellite instability** after carboplatin + paclitaxel + trastuzumab?

1	Chemotherapy	0%
2	Chemotherapy + Pembrolizumab	0%
3	TDM-1	0%
4	Chemotherapy + Lenvantinib	0%
5	Pembrolizumab monotherapy	72.2%
6	Pembrolizumab + Lenvatinib	27.8%
7	Lenvantinib monothterapy	0%
8	Hormonotherapy	0%
9	Abstain	0%

What is the **2nd-line treatment regimen** for metastatic endometrial cancer **HER2 negative with microsatellite instability** after carboplatin + paclitaxel?

1	Chemotherapy	0%
2	Chemotherapy + Pembrolizumab	0%
3	Chemotherapy + Lenvantinib	0%
4	Pembrolizumab monotherapy	78.9%
5	Pembrolizumab + Lenvatinib	21.1%
6	Lenvantinib monothterapy	0%
7	Hormonotherapy	0%
8	Abstain	0%

Is the choice of **2nd-line treatment with Pembrolizumab monotherapy** influenced by the **hormone receptors expression**?

1	Yes	5.6%
2	No	94.4%
3	Abstain	0%

Is the choice of **2nd-line treatment with Pembrolizumab monotherapy** influenced by the **HER2 expression**?

1	Yes	11.1%
2	No	88.9%
3	Abstain	0%

Is the choice of **2nd-line treatment with Pembrolizumab monotherapy** influenced by the **microsatellite instability**?

1	Yes	94.7%
2	No	5.3%
3	Abstain	0%

Is the choice of **2nd-line treatment with Pembrolizumab monotherapy** influenced by the **PD-L1 expression**?

1	Yes	15.8%
2	No	84.2%
3	Abstain	0%

Is the choice of **2nd-line treatment with Pembrolizumab monotherapy** influenced by the **TMB**?

1	Yes	47.4%
2	No	52.6%
3	Abstain	0%

Is the choice of **2nd-line treatment with Pembrolizumab monotherapy** influenced by the **histological subtype**?

1	Yes, only for endometrioid carcinomas	0%
2	Yes, only for serous carcinomas	0%
3	Yes, only for clear cell carcinomas	0%
4	Yes, only for carcinosarcomas	5.6%
5	No	88.9%
6	Abstain	5.6%

Can Pembrolizumab in 2nd-line treatment regimen with ou without Levantinib be modified from 200 mg every 3 weeks to 400 mg every 6 weeks?

1	Yes	38.9%
2	No	50.0%
3	Abstain	11.1%

Should the use of Levantinib combined with Pembrolizumab as part of 2nd-line treatment be routinely started at doses less than 20 mg/day?

1	Yes	47.1%
2	No	52.9%
3	Abstain	0%

What is the **3rd-line treatment** for metastatic endometrial cancer after **1st-line with Carboplatin + Paclitaxel and 2nd-line with Pembrolizumab + Levantinib**?

1	Other chemotherapies regimens	82.4%
2	Chemotherapy + Bevacizumab	0%
3	Hormonotherapy	11.8%
4	Best supportive care	5.9%
5	Abstain	0%

How many lines of chemotherapy should a patient with metastatic endometrial cancer receive regardless of histological subtype or biomarkers?

1	2 lines	0%
2	3 lines	15.8%
3	4 lines	0%
4	As long as it presentes adequate performance status	73.7%
5	Abstain	10.5%
